# Association Between Discrimination and Depressive Symptoms Among Older Blacks: A Pilot Study

**DOI:** 10.1093/geroni/igaa057.973

**Published:** 2020-12-16

**Authors:** Tomorrow Arnold, Courtney Polenick, Frederic Blow

**Affiliations:** 1 University of Michigan, Ann Arbor, Michigan, United States; 2 University of Michigan Medical School, Ann Arbor, Michigan, United States

## Abstract

Racial minority status may be a major source of both acute and chronic stress (e.g., racial discrimination) that contributes to depression among older adults. Indeed, older African Americans experience more psychological distress than their White counterparts largely due to chronic stressors including racism. Yet, little is known about particular aspects of racial discrimination that are most strongly associated with depressive symptoms in middle and later life. The present study is a part of an ongoing pilot project and sought to examine the associations between discrimination experienced in the past month [i.e., perceived ethnic/racial discrimination (PED), everyday discrimination (ED), and heightened vigilance to discrimination (HVD)] and past-month depressive symptoms among Black/African American adults aged 50 to 80 (N = 106). More frequent PED was associated with greater severity in overall depressive symptoms as well as affective and somatic symptoms individually. More frequent ED was associated with greater affect symptom severity only. Increased HVD was associated with greater overall depressive symptom severity, particularly affective symptoms. Discrimination was also differently correlated with specific depressive symptoms. PED was associated with back pain, anhedonia, loss of interest in sex, difficulty breathing, anxiousness, inability to concentrate, and suicidal ideation. Greater ED was associated with anhedonia, chest pain, and feelings of guilt/self-reproach. Higher HVD was associated with chest pain and feelings of guilt/self-reproach. These results suggest that experienced discrimination and attempts to prepare for these experiences may play a role in the presentation of depressive symptomatology and vary by type of symptoms experienced among older Blacks.

